# Increased rates of dispersal of free-ranging cane toads (*Rhinella marina*) during their global invasion

**DOI:** 10.1038/s41598-021-02828-5

**Published:** 2021-12-07

**Authors:** Richard Shine, Ross A. Alford, Ryan Blennerhasset, Gregory P. Brown, Jayna L. DeVore, Simon Ducatez, Patrick Finnerty, Matthew Greenlees, Shannon W. Kaiser, Samantha McCann, Lachlan Pettit, Ligia Pizzatto, Lin Schwarzkopf, Georgia Ward-Fear, Benjamin L. Phillips

**Affiliations:** 1 Department of Biological Sciences, Macquarie University, Sydney, NSW 2109 Australia; 2 College of Science and Engineering, James Cook University, Townsville, QLD 4811 Australia; 3 Rollins College, Winter Park, Florida 32789 USA; 4 School of Life and Environmental Sciences, The University of Sydney, Sydney, NSW 2006 Australia; 5 UMR 241 EIO (UPF, IRD, IFREMER, ILM), Institut de Recherche Pour le Développement (IRD), Papeete, Tahiti French Polynesia; 6 School of Environmental and Life Sciences, University of Newcastle, Callaghan, NSW 2308 Australia; 7 School of BioSciences, University of Melbourne, Parkville, VIC 3010 Australia

**Keywords:** Evolutionary ecology, Invasive species, Tropical ecology

## Abstract

Invasions often accelerate through time, as dispersal-enhancing traits accumulate at the expanding range edge. How does the dispersal behaviour of individual organisms shift to increase rates of population spread? We collate data from 44 radio-tracking studies (in total, of 650 animals) of cane toads (*Rhinella marina*) to quantify distances moved per day, and the frequency of displacement in their native range (French Guiana) and two invaded areas (Hawai’i and Australia). We show that toads in their native-range, Hawai’i and eastern Australia are relatively sedentary, while toads dispersing across tropical Australia increased their daily distances travelled from 20 to 200 m per day. That increase reflects an increasing propensity to change diurnal retreat sites every day, as well as to move further during each nocturnal displacement. Daily changes in retreat site evolved earlier than did changes in distances moved per night, indicating a breakdown in philopatry before other movement behaviours were optimised to maximise dispersal.

## Introduction

The rate at which invasive species spread into new areas determines the timeframe of ecological impact, and is critical for management attempts to buffer those impacts. Thus, understanding factors that influence rates of population expansion is an important challenge. That challenge is complicated by extensive interspecific and intraspecific variation in rates of spread, driven by processes both extrinsic and intrinsic to populations^1,2^. Although the role of environmental and demographic variation is well appreciated, there is also mounting evidence that evolutionary processes contribute strongly to variation in invasion speeds^3,4^. For example, deterministic evolutionary processes on invasion fronts select for high rates of dispersal and population growth, causing invasions to accelerate^5–7^.

Mathematical models predict that individuals at the leading edge of an expanding population will accumulate phenotypic modifications that increase their dispersal ability^8,9^, through a spatial analogue of natural selection called spatial sorting^10^. Laboratory experiments have confirmed that prediction^11–13^, but field studies generally have relied on less direct evidence. Studies of dispersal rate evolution in free-ranging organisms mostly measure either rates of overall population spread^14^, or geographic variation in morphological, physiological and behavioural traits that plausibly influence dispersal ability. For example, individuals at an expanding range-edge might have larger wings or longer legs than do conspecifics in range-core populations^15,16^. In such studies, the links between phenotype, dispersal, and rate of population spread are either assumed or dependent upon correlational data.

We can fill in that knowledge gap, at least partly, by gathering empirical data on rates of dispersal of individuals at range-edges compared to range-core regions; but such studies are rare because it is more difficult to measure movement patterns of free-ranging animals than it is to capture those individuals and measure aspects of their phenotype. Even when dispersal rates of free-ranging animals are measured directly, the data typically are restricted to a small number of populations intended to be representative of different phases of the invasion process^17,18^ or studies at a single site through time^19^. As a result, no published studies have enough population-level replication to robustly identify general patterns in rates of dispersal of free-ranging individuals through the course of an invasion. However, we have now studied the spatial ecology of one iconic invader—the cane toad, *Rhinella marina*—in sufficient detail, in enough places, to fulfil this criterion. Native to South America, toads were translocated to Hawai’i in 1932, and from there to northeastern Australia in 1935—in both cases, to control insect pests of commercial agriculture. In Australia, the toads have since greatly expanded their range across the tropics to the west (moving into increasing hot and arid environments) and along the eastern coast to the south (into cooler climates). The westwards expansion across the tropics has been accompanied by a rapid acceleration in rates of expansion of the geographic range of toads^14^. In the present paper, we collate and analyse raw data from 44 radio-tracking studies on cane toads in three countries (French Guiana, USA and Australia) to explore general patterns of dispersal-relevant behaviour.

## Results

**Figure 1:**
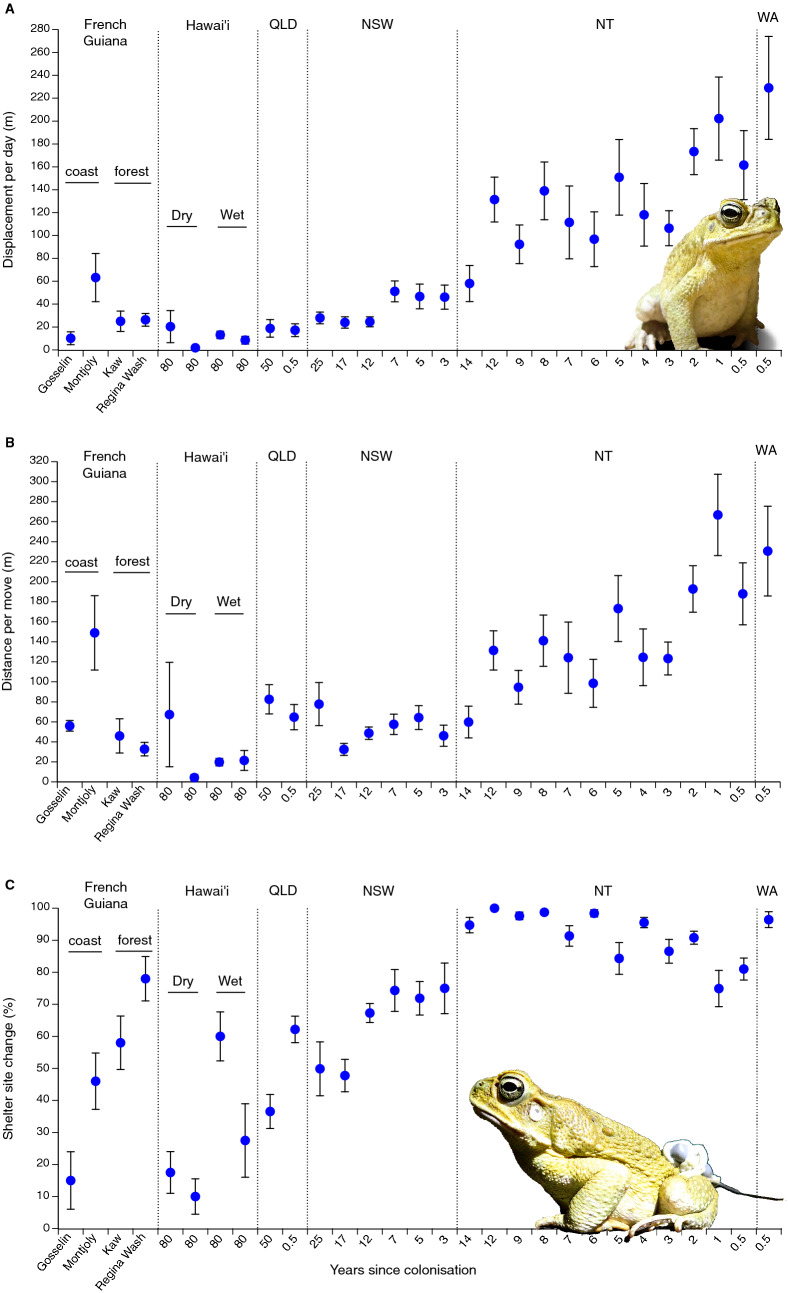
Dispersal traits of cane toads. (**A**) Mean daily displacements by radio-tracked cane toads (*Rhinella marina*) from sites within the species’ native range in French Guiana, and in invaded areas of Hawai'i and Australia (QLD = Queensland; NSW = New South Wales; NT = Northern Territory; WA = Western Australia). (**B**) Mean displacements by radio-tracked cane toads (*Rhinella marina*) on nights when the toads changed diurnal shelter-sites. Data are shown from sites within the species’ native range in French Guiana, and in invaded areas of Hawai'i and Australia (QLD = Queensland; NSW = New South Wales; NT = Northern Territory; WA = Western Australia). (**C**) The proportion of days when radio-tracked cane toads (*Rhinella marina*) changed shelter-sites rather than returning to the same shelter-site on consecutive days. Data are shown from sites within the species’ native range in French Guiana, and in invaded areas of Hawai'i and Australia (QLD = Queensland; NSW = New South Wales; NT = Northern Territory; WA = Western Australia). The Figure plots raw data (means and standard errors) but statistical analyses were based on ln-transformed data. Photographs by J. DeVore.

The mean distances moved by radio-tracked toads varied considerably among sites, from < 10 m per day in some locations in the native range and Hawai’i, through to more than 200 m per day at the invasion front in tropical Australia (Fig. 1a). Overall, dispersal distances were highest in tropical northwestern Australia (Northern Territory and Western Australia: see Fig. 1a, Table 1). Statistical analysis showed a significant link between colonisation history and daily displacements: toads closer to the invasion front (i.e., with lower time since colonisation of the site) moved further each day (Table 1). The distances traversed during displacements (i.e., omitting days when the toad returned to the same shelter-site on successive days) showed a similar pattern (Fig. 1b; Table 1), but with some exceptions to the general trend. For example, mean distances per move were as high in one of the native-range sites (Montjoly Beach) as in locations close to the invasion front in tropical Australia (Fig. 1b). For the third variable that we examined, the proportion of nights that toads changed shelter-sites from one day to the next, considerable variation was evident within the regions where toads were relatively sedentary overall (i.e., French Guiana, Hawai’i, Queensland [QLD], New South Wales [NSW]). By contrast, toads in tropical northwestern Australia showed little variation in this behaviour; most individuals shifted to a new location almost every night (Northern Territory [NT] and Western Australia [WA]: Fig. 1c). Despite that variation within locations, ANCOVA detected an overall link between time since colonisation and proportion of nights when toads moved from one shelter site to another: toads changed shelter-sites more often in invasion-front populations than in areas where the animals had been present for longer periods (Table 1).

**Table 1:** Results of statistical tests on the effects of location and invasion history (years since colonisation of a site) on movement distances and frequencies of radio-tracked cane toads (*Rhinella marina*).

Dependent variable	Covariate	Factor	Statistical parameter	*F*-ratio, df, *P*-value
ln mean distance moved per day	Years since toad arrival at site	Location (random)	Covariate	*F*_1,29.72_ = 17.80, ***P* < 0.001**
ln (1-proportion days with shelter-site changes)	Years since toad arrival at site	Location (random)	Covariate	*F*_1,28.87_ = 25.03, ***P* < 0.0001**
ln distances moved excluding sedentary days	Years since toad arrival at site	Location (random)	Covariate	*F*_1,28.91_ = 21.07, ***P* < 0.0001**
ln mean distance moved per day	Years since toad arrival at site	Region; location	Interaction years*region	*F*_1,649_ = 9.32, ***P* < 0.003**
ln (1-proportion days with shelter-site changes)	Years since toad arrival at site	Region; location	Interaction years*region	*F*_1,648_ = 2.00, *P* = 0.16
ln distances moved excluding sedentary days	Years since toad arrival at site	Region; location	Interaction years*region	*F*_1,649_ = 6.80, ***P* < 0.01**
**Northwestern Australia only**				
ln mean distance moved per day	Years since toad arrival at site	Location (random)	Covariate	*F*_1,10.33_ = 13.66, ***P* < 0.005**
ln (1- proportion days with shelter-site changes)	Years since toad arrival at site	Location (random)	Covariate	*F*_1,12.02_ = 5.59, ***P* < 0.04**
ln distances moved excluding sedentary days	Years since toad arrival at site	Location (random)	Covariate	*F*_1,9.66_ = 17.76, ***P* < 0.002**
**Eastern Australia only**				
ln mean distance moved per day	Years since toad arrival at site	Location (random)	Covariate	*F*_1,8.40_ = 0.40, *P* = 0.54
ln (1-proportion days with shelter-site changes)	Years since toad arrival at site	Location (random)	Covariate	*F*_1,9.35_ = 9.40, ***P* < 0.015**
ln distances moved excluding sedentary days	Years since toad arrival at site	Location (random)	Covariate	*F*_1,5.95_ = 0.48, *P* = 0.51

Columns show dependent variable, covariates, factors, and the statistical parameter for which the final column provides details. Bold font indicates statistically significant results (*P* < 0.05).

An ANCOVA with region as the factor revealed that the effect of invasion history on movement parameters differed among regions for distances moved but not for the proportion of days with changes in shelter-sites (interaction years since colonised*region effects: Table 1). We thus repeated the ANCOVAs on subsets of data from regions that contained sites differing in times since colonisation by cane toads (i.e., northwestern or eastern Australia). All three movement variables were linked to time since colonisation in tropical northwestern Australia. The two distance variables increased closer to the invasion front whereas the proportion of nights when toads changed shelters decreased closer to the front (Table 1). In eastern Australia (QLD plus NSW), neither of the distance variables were significantly linked to time since colonisation, but toads closer to the invasion front shifted shelter-sites more frequently than did conspecifics from long-established populations (Table 1).

## Discussion

Our review of data on free-ranging cane toads reveals a pattern that is largely consistent with theory. In their native range, cane toads are relatively sedentary; a situation that was maintained following introduction to the islands of Hawai’i. The continuous invasion of northern Australia over 80 years is, however, associated with the emergence of highly dispersive phenotypes on the invasion front. Especially in terms of daily distances moved, the geographic pattern is strong (Fig. 1a). Studies on captive-raised offspring of Australian toads have documented significant heritability of dispersal behaviour, suggesting that these are evolved changes rather than phenotypically plastic responses to local conditions^15,20^.

The cline in dispersal rates across northern Australia was first observed some years ago^17,18^, but until a recent telemetry study^21^, the spatial ecology of cane toads within their native range was poorly known. This was an important missing piece of the puzzle, because (despite strong theoretical expectations) the Australian pattern may have been driven not by increased dispersal on the invasion front, but by reduced dispersal in the long-established parts of the range. Comparison with dispersal rates in the native range (based on studies in both beachside and forested habitats^21^) now allows confidence as to the direction of evolution in Australia. Previously published information on dispersal in the native range came from Central America, and were based on studies of a toad species now recognised as *R. horribilis* rather than *R. marina*^22,23^. Based on those reports, the two species may exhibit similarly low vagility in the native range. Although his methods were not directly comparable to ours (reliant on spooling and mark-recapture not telemetry), Bayliss^24^ recorded modest overall dispersal for cane toads in Amazonian Brazil; net displacements were around 30 m per day, within the range we recorded in French Guiana. Overall, available data support the idea that cane toads within their native range are similar to those in long-established Australian populations. Individuals move nomadically, but their dispersal is constrained to relatively slow, short movements. In Puerto Rico (the stepping-stone population between French Guiana and Hawai’i^25^), Carpenter and Gillingham^26^ also reported strong fidelity to water holes in Puerto Rican cane toads.

The translocation of cane toads to Hawai’i in 1932, with subsequent release of thousands of their progeny in sugar-growing areas on all the major islands^25^, has resulted in disjunct populations of toads on the dry (leeward) sides of islands and more continuous distribution on the wetter (windward) sides^27^. In Hawai’i, the toad population did not have thousands of square kilometres of uncolonised suitable habitat through which to invade. Instead, toad populations on Hawai’i experienced strong environmental gradients associated with elevation and between moist and dry sides of each island. Even in areas with high rainfall, porous volcanic soils restrict the availability of moisture and hence make long-distance dispersal risky^27^. The strong spatial heterogeneity and limited vacant habitat on Hawai’i likely explains why cane toads have not evolved increased rates of dispersal there (Fig. 1): spatial heterogeneity directly selects against long-distance movements^28^, and fine-grained spatial heterogeneity can increase the strength of genetic drift such that it overwhelms selection^29^.

The cane toads that were translocated from Hawai’i to Queensland in 1935 encountered a very different opportunity. Here, there was a vast area of suitable habitat to the west of their introduction point. Arid habitats in southwestern Queensland restricted the toads’ geographic spread to the southwest, leaving possible routes of spread in two main directions—northwest through the tropics to the Northern Territory and then Western Australia; and south along the east coast down into New South Wales^14,30^. The toads exploited both of those opportunities, but with different invasion dynamics. The northwestern front accelerated to > 50 km per annum (from an initial spread rate of < 15 km per annum) whereas the southern front moved very slowly (< 10 km per annum^14,31^). In keeping with those rates of population spread, invasion-front toads in northwestern Australia exhibit rapid dispersal whereas southern (NSW) toads do not.

The acceleration in rate of dispersal in northwestern Australia reflects the joint effects of spatial and temporal evolutionary processes^6^. First, the leading tip of the invasion is a region of low conspecific density and populations experience exponential growth. Here, any trait that increases the reproductive rate is favoured. This is standard *r*-selection, and can be seen as a race between genetic variants through time. Second, only the most dispersive individuals make it to the leading edge of the invasion in every generation and so only genes that confer high dispersal rates will be on the invasion front. This is spatial sorting and can be seen as a race between genetic variants through space^10^. On continuous invasion fronts, evolutionary increases in reproductive rate steepen the invasion front and further increase the strength of spatial sorting^9,32^.

That acceleration has not occurred in eastern Australia, either in rates of population expansions^31,33^ or in the movements of individual toads (Fig. 1a). This again likely reflects environmental heterogeneity, albeit in this case manifesting as the latitudinal gradient in temperature and rainfall seasonality that will ultimately place a limit on the toads’ distribution in the south^34^. Low ambient temperatures at night in northern New South Wales appear to limit the rate of spread of these tropical amphibians^35^, and, in keeping with the hypothesis of temperature limitation, the toad front in New South Wales has expanded more rapidly during decades with unusually high ambient temperatures^31^.

The overall rate of daily dispersal of a cane toad (Fig. 1a) is driven by two variables: how far a toad travels when it leaves its previous shelter-site before selecting a new one (Fig. 1b) and the proportion of nights on which a toad moves to a new shelter rather than returning to the previous one (Fig. 1c). Both of these variables shift relative to recency of colonisation of a site (above), but in different ways. Distances travelled were linked more consistently to invasion chronology (Fig. 1a,b) than was frequency of movement from one shelter-site to another. Indeed, changes in shelter-sites were slightly less frequent, not more frequent, close to the invasion front in tropical Australia (Fig. 1c). That non-intuitive pattern likely reflects the toads’ spread into arid regions where moist retreat sites are difficult to find, and hence (as in Hawai’i) favouring re-use of known sites, particularly in the dry season. Toads shifted to new shelter-sites on most nights in some (but not all) populations from throughout the species’ range, including in regions where overall rates of daily dispersal were low (Fig. 1a). Thus, one of the movement parameters that influences dispersal rate (propensity to change shelter-sites frequently) was more variable at a local scale than was the other parameter (distance moved per night). Interestingly, the frequency of changes in shelter-sites decreased closer to the invasion front in tropical northwestern Australia (where the front has accelerated: Fig. 1c) but increased closer to the invasion front in eastern Australia (where the front has not accelerated: Fig. 1c).

The consistently high frequency of shelter-site changes in tropical Australia compared to the ancestral condition (as in coastal French Guiana and Hawai’i: Fig. 1c) suggests that in the course of this invasion, increased rates of dispersal were first achieved by reducing fidelity to specific shelter-sites. An increase in distance moved per night was the second step. Willingness to abandon a previously-used shelter-site requires only a minor behavioural shift. Essentially it is simply choosing not to return home; this may incur little or no cost in the tropical wet-season^36^. Such a shift immediately increases the dispersal rate of the population, but it also exposes other dispersal-relevant traits—such as movement rate, stamina, and path straightness—to spatial sorting. Thus, the progression in displacement behaviour we see in toads may apply generally: the stepwise evolutionary increases in overall dispersal rate during an invasion may be achieved first by a breakdown in site fidelity and only later by optimisation of other dispersal-relevant traits.

Importantly, cane toads also adjust their movement patterns to local conditions, with moist conditions increasing the probability of a toad emerging at night from its diurnal retreat^37^, and moving long distances^38^. The spatial distribution of local resources—such as areas for sheltering, feeding, rehydrating and breeding—also influences movements^21,39^. Thus, the behaviours seen in invasion-front cane toads (i.e., frequent changes in shelter-sites, and long-distance displacements) are manifested in all populations of cane toads, even in the native range—but only by some individuals, some of the time^21,36^. In the course of their international diaspora, some populations of cane toads—those in northwestern Australia—have shifted to constitutive expression of these previously-facultative traits. All cane toads are capable of rapid dispersal, but exhibit those rates only under specific conditions^40,41^. That scenario may be common; for example, Komodo dragons have profound capacities for navigation and movement, despite most individuals never leaving the valley in which they were born^42^. Given sufficient evolutionary pressure, these latent abilities can become the predominant phenotype: at the tropical Australian invasion front, all toads disperse rapidly, and do so as long as conditions allow it.

Future work could usefully examine geographic variation in other traits that influence overall rates of dispersal. For example, one of the most efficient ways to disperse long distances is to keep moving in the same direction night after night. Cane toads at the invasion front in tropical northwestern Australia tend to move in this way, and this path straightness is heritable^20^. It is easy to imagine other behavioural traits that would enhance rates of dispersal, such as a preference for moving along open linear corridors rather through dense vegetation^40^. More detailed studies of cane toads at different stages of the invasion process could explore routes as well as rates of dispersal (e.g., avoidance of dispersal barriers), and the timing and abiotic correlates of dispersal behaviour (what conditions of darkness and moisture elicit emergence at dusk?).

To evaluate generalities in the evolutionary processes driving rapid evolution during biological invasions, it would also be of interest to examine other examples. Is it generally true, for example, that evolutionary innovation is first driven by changes in behaviour, before morphological and physiological traits evolve^43–45^? In many cases, some level of behavioural shift is required to impose selection on non-behavioural traits. For example, soapberry bugs (*Leptocoris tagalicus*) likely exhibited at least a partial switch in hostplant preference prior to evolving longer mouthparts that enhanced the bugs’ ability to feed on the invasive plant^46^, and lizards (*Anolis carolinensis*) presumably shifted to higher perches following invasion by competitors before evolving morphological adaptations (larger toepads) that facilitate arboreal activity^47^. Analogously, cane toads appear to have evolved more frequent shifts among refuge-sites prior to evolving enhanced rates of daily dispersal.

In summary, our data on cane toads provide strong evidence for shifts in dispersal behaviour in the course of a biological invasion. Importantly, we have measured actual rates of dispersal rather than relying upon putative links between an individual’s phenotype and its likely rate of dispersal. Our data reinforce the emerging conclusion that we need to incorporate evolutionary thinking into our approach to wildlife management; the speed and magnitude of shifts in important traits (such as those driving dispersal propensity) may be high enough that we ignore them at our peril.

## Methods

### Study species

**Figure 2:**
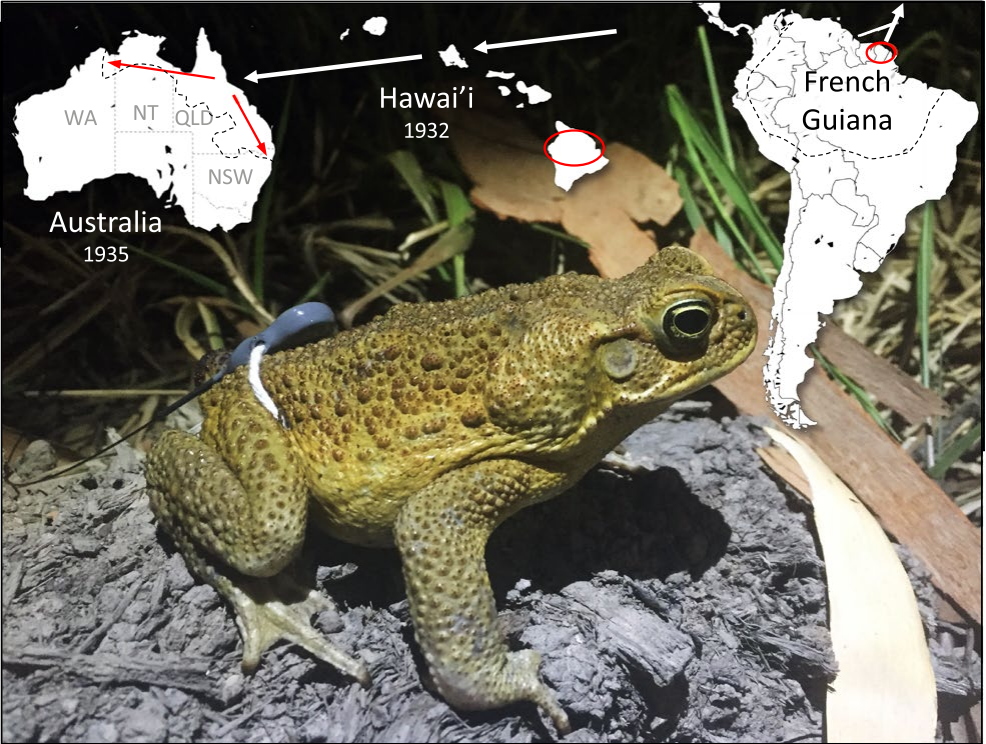
Cane toad (*Rhinella marina*) with radio-transmitter attached to waist belt. Photograph by Matthew Greenlees. Cane toads were exported from their native range in the Guianas to the Caribbean, before being taken from Puerto Rico to Oahu, Hawai’i. Toads were further spread across the Hawai’ian islands of Kauai, Molokai, Lanai, Maui, and Hawai’i (i.e., “the Big Island”, where tracking data were collected) from 1933 to 1935. Toads from Oahu were also transported to Queensland (QLD), Australia. Here we have depicted the regions in which toad movements have been radio-tracked in red. Within Australia, the red arrows indicate the two routes of spread from the original introduction site (northwestern and eastern), and the black dotted line indicates the extent of their current distribution.

The cane toad (*Rhinella marina*; *Bufo marinus* in earlier literature) is among the largest anuran species (exceptionally, to > 1 kg in mass), and is native to northern South America east of the Andes^48^ (Fig. 2). Members of the same clade from west of the Andes, previously allocated to *R. marina*, are now recognised as a separate species, *R. horribilis*^48^. Bufonid anurans are capable of sustained locomotion^49^, but most field studies of toads report sedentary behaviour interspersed (in some species) with long seasonal migrations^50–52^. Studies in Australia suggest that newly-metamorphosed toads are highly philopatric to the natal waterbody until they grow large enough to resist desiccation and thus, can move into the more arid surrounding habitat^53^. Adult cane toads are primarily inactive by day (but see Pettit et al.^54^ for exceptions), within cool moist retreat-sites^37^. The animals emerge at night to forage, rehydrate and breed^27,40^. Because dispersal occurs by night, the linear distance between locations of diurnal retreat-sites used on successive days offers a simple metric of dispersal.

Cane toads are a prolific invasive species, translocated to many places around the world in attempts to control insect pests in commercial sugarcane plantations. These toads were taken from French Guiana and Guyana to sugar-cane plantations in Puerto Rico (via Martinique, Barbados, and/or Jamaica) in the early nineteenth century, and 150 individuals from Puerto Rico were translocated to Hawai’ian plantations in 1932^25^. Three years later, 101 toads from Hawai’i were brought to northeastern Queensland, Australia, and their progeny released in sugar-growing areas over much of the eastern Queensland coast. The toads failed to control insect pests^55^ but dispersed rapidly. Rates of invasion accelerated from around 10 km per annum to > 50 km per annum as the toads spread westward through Queensland and the Northern Territory into Western Australia^14,38,56^, but spread rates remained low as the toads moved south into cooler regions of southern Queensland and New South Wales^30,31,33^.

### Methods

We compiled original data from 44 radio-tracking studies (total of 29 sites, 650 toads; *n* = 3–75 toads tracked per site) that we conducted using consistent methods. Adult cane toads were captured in the wild, fitted with small radio-transmitters on waist belts (Holohil model PD-2, 2.5 gm, < 3% of toad body mass), released at the site of capture, and then relocated on a daily basis (where possible) for at least 5 days thereafter. We did not include data from spool-tracking studies^24,57^, or from studies with longer intervals between relocations^39^. Most of the studies were conducted by members of the same research group, over a 16-year period (2005 to 2020). In some cases, studies were conducted at the same sites several years apart, and hence with different times since initial colonisation by toads. All studies were conducted during weather conditions that facilitate toad activity (warm, with at least intermittent rain^37,38^), and we excluded data from toads that had been subjected to experimental treatments (such as translocation, captive rearing, or removal of parasites^58,59^) that might change their dispersal behaviour.

From the available data, we extracted information on two variables: (1) mean distance moved per day (from one diurnal shelter site to the next, including occasions when the same shelter was used) and (2) the proportion of nights that the toad changed diurnal retreat-sites (i.e., was found in different shelters > 5 m apart on successive days). From these two variables we also calculated (3) daily distance moved in cases where the toad changed shelter-sites (i.e., omitting zeros for times when toads did not change shelter-sites from one day to the next).

### Ethical note

This paper is based on a collation of data from previous research; no additional animals were handled in the course of the current work. In the original studies, all experimental protocols were approved by institutional committees (University of Sydney Animal Care and Ethics Committee; James Cook University Animal Care and Ethics Committee) and were conducted in accordance with all relevant guidelines and regulations, including the ARRIVE guidelines.

### Analysis

We classified each study site in terms of time since invasion by toads (based on historical records^14,60^). Table 2 provides data on study site locations, the seasonal timing of radio-tracking, and the number of toads tracked. We ln-transformed the dependent variables to improve normality; for proportion of shelter-site changes our statistical analysis used ln (1-proportion of changes). To examine the predicted increase in dispersal-rate attributes close to the invasion front, we used ANCOVA with the dispersal parameter (e.g., ln daily distance travelled) as the dependent variable, years since invasion of the site as a covariate, and population identity as a random factor (to allow for measures on multiple toads within a single population). For the purposes of this analysis, we allocated the four native-range sites an arbitrary “age since colonisation” of 100 years (i.e., marginally longer than any of the invaded sites). To evaluate whether or not regions differed in the effects of invasion history (time since colonisation) on movement parameters, we used an ANCOVA with region as the factor and invasion history as the covariate, plus the interaction term between region and invasion history. We then conducted ANCOVAs separately on data for the two regions within which sites with a range of invasion histories were sampled (eastern Australia and tropical northwestern Australia).

**Table 2:** Sites at which cane toads (*Rhinella marina*) were radio-tracked, showing locations, colonisation histories, seasonal timing of radio-tracking studies, number of toads tracked per site, and papers describing these projects.

Study site category	Study site locations	Latitude and longitude	Years since colonisation	Timing of study	N toads tracked	Authority
French Guiana coastal	Gosselin Beach	4.89°7ʹS, 52°25ʹW	> 100	Oct–Nov 2017	10	21
French Guiana coastal	Montjoly Beach	4.91°33ʹS, 52°26ʹW	> 100	Oct–Nov 2017	10	21
French Guiana rainforest	Regina Wash Rainforest	4.29°44ʹS, 52°22ʹW	> 100	Oct–Nov 2017	10	21
French Guiana rainforest	Kaw Rainforest	4.64°37ʹS, 52°30ʹW	> 100	Oct–Nov 2017	3	21
Hawai'i Dry-side	Mauna Kea golf course, Hawai'i	19°59ʹN, 155°49ʹW	80	Jun–Jul 2015	10	27
Hawai'i Dry-side	Waikola Beach resort, Hawai'i	19°56ʹN, 155°47ʹW	80	Jun–Jul 2015	10	27
Hawai'i Wet-side	23 km south of Hilo, Hawai'i	19°39ʹN, 155°04ʹW	80	Jun–Jul 2015	10	27
Hawai'i Wet-side	University of Hawai'i campus at Hilo	19°42ʹN, 155°04ʹW	80	Jun–Jul 2015	10	27
Queensland	Tabletop Station, Townsville	19°23ʹS, 146°26ʹE	50	Feb–May 1996	18	37
Queensland	Heathlands Ranger Station, Cape York Peninsula	11°46ʹS, 142°36ʹE	0.5	Apr–May 1992; Jan–Feb 1993	25	36
Northeastern NSW	Rocky Creek Dam and Mt Warning	28°23ʹS, 153°16ʹE	25	Sep–Oct 2012	16	61
Northeastern NSW	Doubleduke Forest	29°8ʹS, 153°12ʹE	17	Nov–Dec 2020	45	62
Northeastern NSW	Bosches Road, Lewis Lane, Mt. Nardi	28°32ʹS,153°17ʹE	12	Oct 2012–Feb 2013	75	61
Northeastern NSW	Brooms Head	29°36ʹS, 153°20ʹE	7	Oct 2015–Jan 2016	21	41
Northeastern NSW	Doubleduke Forest	29°8ʹS, 153°12ʹE	5	Nov–Dec 2020	28	62
Northeastern NSW	Doubleduke Forest	29°8ʹS, 153°12ʹE	3	Nov–Dec 2020	5	62
Northern Territory	Leaning Tree Lagoon	12°42ʹS, 131°25ʹE	14	Aug–Nov 2016	19	63
Northern Territory	Leaning Tree Lagoon	12°42ʹS, 131°25ʹE	12	Aug–Nov 2016	11	63
Northern Territory	Adelaide River floodplain	12°38ʹS, 131°19ʹE	9	Jan 2014–Apr 2014	39	64
Northern Territory	Adelaide River floodplain	12°38ʹS, 131°19ʹE	8	Jan 2013–Mar 2013	28	19
Northern Territory	Adelaide River floodplain	12°38ʹS, 131°19ʹE	7	Jan 2012–Apr 2013	22	19
Northern Territory	Adelaide River floodplain	12°38ʹS, 131°19ʹE	6	Nov 2010–Mar 2011	21	19
Northern Territory	Adelaide River floodplain	12°38ʹS, 131°19ʹE	5	Nov 2009–Mar 2010	21	19
Northern Territory	Adelaide River floodplain	12°38ʹS, 131°19ʹE	4	Nov 2008–Mar 2009	26	19
Northern Territory	Adelaide River floodplain	12°38ʹS, 131°19ʹE	3	Nov 2007–Mar 2008	34	19
Northern Territory	Adelaide River floodplain	12°38ʹS, 131°19ʹE	2	Nov 2006–Mar 2007	51	19
Northern Territory	Adelaide River floodplain	12°38ʹS, 131°19ʹE	1	Nov 2005–Mar 2006	22	19
Northern Territory	Adelaide River floodplain	12°38ʹS, 131°19ʹE	0.5	Feb–Apr 2005	22	40
Western Australia	Oombulgurri	15°10ʹS, 127°50ʹE	0.5	Nov 2014–Feb 2015	28	65

## Data Availability

Data are available in the Dryad Data Repository https://doi.org/10.5061/dryad.tb2rbp021.
